# A Quasi-Experimental Study: Social Support in Group Prenatal Care’s Impact on Postpartum Depression in Black and Hispanic Women

**DOI:** 10.3390/ijerph22071046

**Published:** 2025-06-30

**Authors:** Keisha A. Robinson, Tarnisha Ebony Hemphill, Robert O. Atlas

**Affiliations:** 1 School of Nursing, University of Maryland, Baltimore, MD 21201, USA; 2 George Washington University, Washington, DC 20052, USA; tarnisha.hemphill@gwu.edu; 3 School of Medicine, University of Maryland, Baltimore, MD 21201, USA; ratlas@mdmercy.com

**Keywords:** Black women, Hispanic women, group prenatal care, postpartum depression

## Abstract

Depression is a widespread mental health condition that affects millions of women globally. In the United States (U.S.), more than half of maternal mental health-related deaths occur during the postpartum period, making it the leading cause of mortality during this time. This urban U.S. single-site quasi-experimental study aimed to evaluate the effectiveness of social support integrated into group prenatal care as an intervention for postpartum depression. The study employed a dual methodological approach, combining prospective participant recruitment with a retrospective analysis of medical records. It compared the Edinburgh Postnatal Depression Scale (EPDS) scores from group prenatal care to those from traditional individualized prenatal care, specifically focusing on Black and Hispanic women. In all, 200 postpartum women participated in the study, comprising (n = 100) group prenatal care and (n = 100) traditional individualized care. Most participants were Black (97%), with an average age of 26.8 years (SD = 5.9). At six weeks postpartum, 97% of the participants underwent depression screening, which indicated a mean EPDS score of 3.79 (SD = 4.7). Among the participants, 25% exhibited mild to moderate postpartum depression, while 3% experienced severe depression. No significant differences were observed between the models of care in terms of total scores (T = 2.0, *p* = 0.46) or score ranges (χ^2^ = 5.8, *p* = 0.12). It is noteworthy that no severe cases of depression were identified within the group prenatal care model. Suggesting group prenatal care may still benefit Black and Hispanic women in urban areas with a history of anxiety or depression.

## 1. Introduction

Postpartum depression affects approximately 14% of women in the United States [1,2]. Women with a history of depression diagnosed either before or during pregnancy are at an increased risk of developing postpartum depression (PPD), a component of depression [2]. Typically PPD symptomology occurs within the first four to six weeks but may develop up to one year after childbirth [3]. PPD is characterized by persistent feelings of sadness, self-doubt, hopelessness, and exhaustion, as well as an overwhelming sense of despair that extends beyond the typical “baby blues” [4]. In some cases, symptoms of PPD may escalate, leading women to experience thoughts of harming themselves or their newborn [4].

Extensive research indicates that Black and Hispanic women are more than twice as likely to experience PPD compared to their White counterparts [5,6,7]. The prevalence of PPD among Black women is 43.9%, Hispanic women 46.8%, while White women experience PPD at a significantly lower rate of 31.3% [5,6,7]. A known key driver of the disproportionate prevalence of PPD experienced by Black and Hispanic women is living within an urban environment. Research indicates that Black and Hispanic pregnant women living in urban areas plagued by property damage and vacant housing face a higher risk of developing maternal mental health issues, particularly PPD [8]. Additionally, pregnant women in urban environments often lack access to protective factors against depression, such as green spaces that encourage social interaction and foster supportive relationships with friends and family [9,10]. Instead, they suffer from higher levels of PPD, worsened by significant environmental noise and chemical pollution from traffic and high population density, which can lead to impersonal relationships associated with low social cohesiveness [8,9,11].

Additional predisposing environmental factors experienced by Black and Hispanic pregnant women include structural racism, implicit bias, limited access to culturally competent mental health services, and a significant risk of experiencing an adverse childbirth outcome [5,12]. These challenges act as psychological and physical stressors, significantly increasing the risk of developing PPD [5,9,12]. Moreover, Black and Hispanic women residing in urban environments are more likely to experience a diminished rapport with their healthcare providers, primarily attributed to the time constraints inherent in healthcare appointments [5,13]. This challenge adversely impacts the ability of Black and Hispanic postpartum women to communicate their symptoms adequately, thereby lowering the probability that healthcare providers will identify instances of PPD accurately [5,13]. Furthermore, when clinicians are uninformed about the varying cultural symptomology of mental illness among a vast population, mental illness is often undiagnosed, misdiagnosed, or misunderstood [13].

Besides environmental factors, stigma impacts the underdiagnosis and undertreatment of PPD among Black and Hispanic women [7,14]. Cultural stigma significantly impacts the willingness of women to seek support from family members and partners when postpartum depression symptoms arise. Stigma creates barriers to open communication and emotional assistance [4,12]. Furthermore, once professional mental health services are accessed, cultural barriers to effective treatment persist. Black and Hispanic postpartum women are identified in the literature as less likely to engage in medical management forms of treatment [15]. Common reservations concerning antidepressant use include fear of addiction and potential side effects on themselves or their babies through breast milk transmission [15]. Instead, studies show that postpartum women belonging to minority groups often turn to informal sources of support, such as their social networks, unlike White postpartum women [7,15,16]

The sequela of PPD also disproportionally affects Black and Hispanic persons [1]. The last decade has seen a notable rise in suicidal ideation and self-harm among postpartum Black women in the U.S. [1]. The Maternal Mortality Review Committees identified maternal mental health conditions as one of the leading causes of preventable maternal deaths during the first year postpartum and found that PPD accounts for 23% of deaths by suicide [17]. PPD affects not only postpartum women but also has a proven negative impact on personal, family, and child developmental outcomes [18,19]. PPD among Black and Hispanic postpartum women profoundly disrupts maternal–infant bonding, strains family relationships, and perpetuates cycles of health disparities [5,19,20].

### Mitigating Postpartum Depression

Numerous studies indicate that mental health support for expectant women can be enhanced at the healthcare organizational level through the implementation of Group Prenatal Care (GPC) [7,21,22]. The GPC model, facilitated by healthcare professionals, often midwives, is an alternative to individualized prenatal care. GPC involves a group of pregnant women with similar due dates and has been acknowledged as a practical approach for delivering essential prenatal care to expectant parents and increasing knowledge about pregnancy and the postpartum period [5,7,21]. The informational support offered to women in GPC has been shown to lead to better childbirth outcomes, particularly by significantly reducing preterm birth rates [23]. This improvement is especially notable among Black women, who are at a higher risk for preterm birth compared to Hispanic and White women [5,23]. The social support component of GPC is thought to be beneficial for both Black and Hispanic women. It is believed to enhance self-esteem and reduce feelings of self-blame and shame, which in turn positively impact maternal mental health outcomes, specifically PPD [23]. Social support refers to the psychological and material resources a social network provides to help individuals cope with stress and adversity [24]. Within the context of GPC, social support encompasses the experience of prenatal care, which involves acquiring new knowledge, establishing positive relationships with healthcare providers, and forming supportive peer connections [25].

The hallmark of GPC is its positive outcomes, including higher attendance rates for prenatal appointments, lower rates of preterm births, and fewer instances of low birth weight; however, it rarely measures postpartum depression (PPD), specifically in Black and Hispanic populations [26]. A comprehensive literature review of peer-reviewed articles examining “GPC and PPD in Black and Hispanic women in the United States” over the past decade revealed a lack of relevant findings within our academic databases. However, by broadening our search parameters to encompass all racial and ethnic groups and international studies, we identified several articles of considerable relevance to the topic.

A recently published systematic review of the literature revealed that, when targeted mental health therapies were embedded within GPC, the likelihood of screening positive for PPD was reduced [21]. This was further demonstrated in a later study involving 550 Ethiopian women; the combination of GPC social support and psychoeducation therapy was shown to significantly reduce postpartum depression scores on the Patient Health Questionnaire-9 [27]. The researchers suggest that, although embedding psychoeducation therapy within their GPC model yielded beneficial effects, the presence of social support was most protective against PPD (AOR = 0.04, 95% CI = 0.01–0.15) [27]. Similar PPD findings were observed in women seeking prenatal care with opioid use disorders. Group prenatal care was combined with cognitive behavioral therapy for substance use disorder and was found to reduce PPD symptomatology based on Edinburgh Postnatal Depression Scale responses [28]. No predictor analysis was conducted to determine which treatment, GPC or cognitive behavioral therapy, was most protective of PPD [28].

However, inconsistent findings have emerged from various studies regarding the effectiveness of GPC as a singular intervention for mitigating PPD among women in the United States [23]. Earlier research involving adolescent women at high risk for PPD demonstrated that social support within the GPC framework could alleviate symptoms of PPD [28]. Additionally, GPC has been recognized as an effective non-pharmacological approach for reducing both perinatal and postnatal depressive symptomatology [29].

There is consistent evidence that GPC can also serve as a mitigator of depression symptomatology during the postpartum period, particularly when combined with other therapies. However, further investigation is necessary to elucidate the relationship between social support provided through GPC as the primary intervention and PPD. While some studies suggest that GPC may help alleviate symptoms of PPD, the overall effectiveness of GPC for this condition remains inconsistent in the existing literature. Therefore, this study aims to explore the relationship between group prenatal care and Edinburgh Postnatal Depression Scores among Black and Hispanic postpartum women.

## 2. Materials and Methods

### 2.1. Study Design, Setting, and Population

This quasi-experimental investigation employed a convenience sampling methodology that integrated both prospective recruitment and retrospective review of medical records to assemble two distinct cohorts of women seeking prenatal care from an obstetric practice in Baltimore City, Maryland. The study site is notable because over half of maternity care is provided to Medicaid recipients who require additional public support services. The site’s demographics are such that over 80% of pregnant women seeking care identify as either Black or Hispanic and primarily reside in Baltimore City or its adjacent counties. The residential environments of potential participants exhibit factors that may potentiate PPD. Notably, Baltimore City struggles with a significant amount of vacant housing and limited green space. Baltimore City has the highest rate of vacant residential properties at 7.2%, with less than 10% of its land devoted to green space and an average annual household income of 59 K [30,31,32]. Participant inclusion criteria consisted of non-randomized Black and Hispanic birthing persons who, after informed consent, self-selected to receive either individualized prenatal care (IPC) or GPC at the recruiting obstetrical practice. The exclusion criteria for the study aligned with exclusionary criteria for GPC, which included specific medical conditions known to increase the risk of preterm labor, preterm birth, and adverse neonatal outcomes. Exclusionary medical conditions at our recruiting site included: 1. autoimmune disorders; 2. malignancy requiring treatment during pregnancy; 3. active seizure disorder; 4. cardiac conditions warranting care by a cardiologist; 5. uncontrolled thyroid disorder; 6. fetal abnormalities; and 7. human immunodeficiency virus.

### 2.2. Data Collection

The recruiting obstetrical practice’s standard of care requires that all birthing persons seeking care at the practice first have an intake visit with the office-registered nurse. During the intake visit, a medical and obstetrical history is collected. If the birthing person’s medical history aligns favorably with the GPC’s inclusion/exclusion criteria, the intake registered nurse provides information and literature on GPC and IPC. Shortly after the intake visit, but before the initial obstetrical visit, women self-enroll in either GPC or IPC.

To reduce bias, prospective recruitment strategies included the first author, who did not provide GPC or IPC at the recruitment site, to be physically present to verbally invite women receiving IPC and those engaged in the GPC to participate in the study. During the verbal recruitment process, participants were provided with comprehensive information regarding the study’s objectives, the nature of their involvement, the anticipated duration of the study, potential risks and benefits, and assurances regarding maintaining privacy and confidentiality. Most importantly, participants were informed that their involvement in the study was entirely voluntary. Upon agreeing to participate, written consent was duly secured.

The demand for GPC among birthing persons quickly surpassed the expectations of the OB practice. Instead of adhering to the planned biweekly schedule for GPC sessions, the frequency escalated to 4–5 sessions per week, selectively arranged based on the availability of trained GPC midwife providers. This significant expansion and complex scheduling hindered our ability to ensure the presence of a research team member knowledgeable about the study but not directly involved in providing GPC or IPC at the site, specifically the study’s first author. In contrast, recruiting women for IPC proved to be more straightforward and allowed for greater flexibility regarding the first author’s availability for consent, with approximately 26 obstetrical sessions conducted weekly. As a result, the number of prospectively recruited IPC participants greatly exceeded that of GPC participants, prompting our research team to seek IRB approval for a retrospective chart review approach. The retrospective chart reviews allowed us to maintain integrity while capturing GPC data comprehensively.

In all, 105 charts were reviewed, 60 GPC and 45 IPC. This review took place from January 2025 to March 2025. It included all pregnant Black and Hispanic women who participated in either the GPC or IPC and met the predetermined inclusion criteria for the study. The Obstetrical Care Navigator at the recruitment site conducted the initial medical record abstraction using EPIC electronic health records (EHRs) to produce a report of active pregnancy episodes within the obstetrical practice, identifying women who received GPC and IPC and gave birth within a similar timeframe. The first author was the second reviewer of the medical records retrieved from EPIC EHRs. Utilizing the International Classification of Diseases, 10th revision (ICD-10) codes alongside manual chart audits, the first author ensured that the women enrolled in IPC met the study’s inclusion and exclusion criteria. The use of ICD-10 codes also assisted with the identification of both GPC and IPC participants who received a six-week postpartum exam. This approach facilitated moderate control over selection biases and extraneous variables, including environmental factors such as adverse weather conditions, seasonal illnesses, COVID-19 spikes, and trending social stressors.

The data consisted of prenatal laboratory results, birth records, and postpartum data from enrolled women. No personal identifying information, such as names, birthdays, or Social Security numbers, was collected. The study was approved by the Institutional Review Board at XX Medical Center and granted exempt status.

### 2.3. Intervention

In alignment with the Centering Healthcare Institute’s model of GPC, the model encompasses three primary components: health assessment, education, and support [33]. Women who participated in the GPC model were organized into groups of eight to twelve individuals, each composed of women at similar stages of gestation. The program included ten biweekly meetings spanning the first and third trimesters of pregnancy, fostering an environment that promoted active engagement and autonomy. Participants were encouraged to monitor their vital signs under the guidance of trained personnel, which enhanced their involvement in the care process.

The support aspect served as the intervention GPC and was structured around four dimensions: tangible support, informational support, affectionate support, and network support. Tangible assistance included provisions for free transportation and complimentary groceries. Informational support was delivered through comprehensive educational sessions covering a spectrum of topics pertinent to pregnancy, such as common physical discomforts, nutritional guidance, exercise recommendations, prevalent comorbidities, the birthing process, newborn care, postpartum care, and mental health issues, particularly postpartum depression.

Affectionate support was facilitated by inviting spouses, domestic partners, or identified support persons to participate in GPC sessions alongside the pregnant women. Crucially, the GPC framework enabled pregnant women to establish connections and forge a supportive network with peers who shared similar objectives for achieving a healthy pregnancy and childbirth experience, thus providing network support.

### 2.4. Measure

The 10-item Edinburgh Postnatal Depression Scale (EPDS) was utilized in this study to assess depression symptomology in women between six and eight weeks postpartum. The EPDS is a widely recognized and self-administered instrument that has undergone extensive validation across diverse racial and ethnic cohorts [34,35]. It has been translated into 57 languages, enhancing its accessibility and applicability in cross-cultural contexts. Furthermore, EPDS demonstrates utility in evaluating depressive symptoms during the prenatal period, highlighting its versatility in maternal mental health research. The EPDS exhibits strong internal consistency and reliability for up to one year postpartum, with a Cronbach’s alpha of 0.84 [34,35]. The EPDS scale comprises ten items that assess depressive symptoms over the past week, with each item offering four response options, scored from 0 to 3. The total score can range from 0 to 30; scores of 10 or higher suggest the possibility of postpartum depression [34]. The EPDS also features four distinct ranges: none/minimal (0–6), mild (7–13), moderate (14–19), and severe (19–30) [36]. Women with more than one missing response on the EPDS were excluded from scoring due to a lack of guidance from the developers regarding scoring in the presence of missing data.

### 2.5. Sample Size Determination

A priori power analysis indicated that a total sample size of 102 women, with 51 individuals allocated to each group, would be necessary to effectively assess the mean differences in postnatal depression scores between the two independent cohorts. This sample size was determined to achieve a medium Cohen’s D effect size of 0.5, an alpha of 0.05, and a 1-beta of 0.80, thereby ensuring the statistical rigor of the findings between participants receiving GPC and those receiving IPC.

### 2.6. Analysis

Data were evaluated using descriptive statistics with IBM SPSS version 27. Frequency and proportions were used to describe participants’ demographic characteristics and item responses to the EPDS. Pearson’s correlations and chi-squared analysis explored the relationships between participants’ demographics, pregnancy characteristics, and overall EPDS scores. An independent *t*-test assessed the mean differences between GPC and IPC EPDS score ranges and total scores.

## 3. Results

### 3.1. Characteristics of Participants

Two hundred postpartum women were recruited, with 100 participants enrolled in GPC and 100 in IPC. Out of the 200 participants, 1 prospective GPC participant did not return for postpartum care and thus did not receive PPD screening, while another prospective participant in IPC transferred their care before giving birth. Furthermore, 97 percent of the participants were Black, and 3% were Hispanic; none identified as both Black and Hispanic, with most (68.2%) residing in Baltimore City (Table 1). Eighty-two percent were unmarried, with a mean age of 26.8 years (SD = 5.9), and they gave birth at a gestational age of 38.6 weeks (SD = 1.7). More than half (62%) gave birth via spontaneous vaginal delivery. Forty percent were first-time mothers, with a mean postpartum hospital stay of 2.7 days (SD = 1.9). Less than 20% of participants reported having a medical history of either anxiety, depression, or both. Collectively, participants had a mean EPDS score of 4.0 (SD = 4.6), with 13.8% having a total score greater than 10, indicating a risk for PPD. When examining PPD ranges in this cohort, 24% had mild to moderate PPD, and 3% had severe PPD.

### 3.2. EPDS Responses

Participants’ responses to individual EPDS items varied (Table 2). More than eighty percent of postpartum women were able to see the funny side of a situation and look forward to the future with excitement. However, some women (20%) experienced some difficulty with feelings of enjoyment when queried about the future. Slightly more than half (56.1%) of participants never blamed themselves unnecessarily when things went wrong, 41.5% occasionally blamed themselves, and 2.5% blamed themselves most of the time. Additionally, at times, women experienced feelings of anxiety (35%), while (5.1%) worried and were anxious for no good reason very often. Notably, 48% of women had mild to moderate difficulties with coping (34.9%), mild to moderate feelings of being miserable (3.5%), and experienced suicidal ideation.

### 3.3. Association Between Postpartum Depression and Participants’ Characteristics

When examining the relationships between PPD scores and participants’ demographics and characteristics, only the participants’ age was shown to have a significant relationship with EPDS scores. While well-established correlations frequently observed in women’s health literature were replicated, age and marital status (r = 0.196, *p* < 0.001), as well as marital status and history of previously giving birth (r = 0.142, *p* = 0.05), were also significant. Notably, no relationship was observed between the model of prenatal care received and EPDS total scores (T = 2.09, *p* = 0.461).

Table 3 provides a granular analysis of the prevalence of moderate to severe levels of depression. Participants within the age range of 21–26 years and those in the 26–30-year age group had cases of severe depression. Regarding the social support intervention within GPC, no significant difference was observed in the EPDS ordinal categories between the GPC and IPC cohorts (χ^2^ = 5.8, *p* = 0.12), as shown in Table 4. Notably, while the difference was not statistically significant, none of the women enrolled in the GPC displayed an EPDS score in the severe range. Despite this insignificant finding, GPC showed practical benefits, particularly assisting women in averting severe PPD.

## 4. Discussion

Despite recruiting an equivalent number of participants to each cohort, at first glance, there appears to be an underrepresentation of Hispanic women participants. According to state data, both Hispanic men and women comprise approximately 7.8% of the recruitment city’s population [37]. Therefore, this single study comprehensively represents the Hispanic women’s population residing in Baltimore City and its adjacent counties. However, it may not demonstrate generalizability to other urban environments with larger Hispanic populations. Furthermore, demographic and birth data for both Black and Hispanic participants closely align with childbirth statistics collected by the study site’s state health department [38].

The findings from the individual analysis of EPDS item responses are also consistent with the existing literature, specifically in terms of PPD symptomatology consisting of feelings of anxiety and behaviors of self-blame [39]. However, compelling research indicates that Black women in the postpartum period often report physical or somatic symptoms, such as unexplained aches, pains, fatigue, and gastrointestinal issues, when experiencing PPD [40]. These symptoms are not addressed in the EPDS. This gap in understanding the variations in PPD symptomology may have hindered our capacity to identify, diagnose, and, most importantly, treat postpartum depression effectively.

Additionally, the total rate of mild to severe PPD among women in both the GPC and IPC groups in this study was 24.5%, remarkably lower than the PPD prevalence observed in Black women (43.9%) and Hispanic women (46.8%) [5,6,7]. This finding may highlight the limitations of relying solely on the EPDS for identifying PPD. Additionally, it could suggest that there are confounding factors within the community that may act as protective influences against PPD.

Finally, although group prenatal care did not demonstrate a significant change in PPD outcomes, social support within GPC did provide some level of support for maternal mental health during the postnatal period. In this study, GPC enrolled the highest percentage of unmarried primigravids with a history of depression and/or anxiety from inner-city Baltimore, who are burdened by residential factors that exacerbate depression. Despite this, no participant enrolled in GPC experienced PPD symptomology within the severe range, leaving our research team to believe that social support within GPC does yield some benefit toward alleviating PPD symptomology in urban Black and Hispanic women.

### 4.1. Implications

Our study revealed an association between maternal age and depressive symptomatology; however, this relationship does not appear to be restricted to a specific age group. Intriguingly, in our study, the youngest subgroup, specifically teenagers, demonstrated lower rates of mild, moderate, and severe depression when compared to their counterparts in the 21–30-year age category. This finding does not align with the literature, which suggests teenagers are most at risk for PPD when compared to other age groups [41]. Our findings underscore the necessity for further research into how women perceive support, as it may differ, especially between teenagers and adults. Additionally, research should examine the potential confounding effects of enhanced social support through state and local government initiatives to assist teenage mothers. If such confounding influences exist, the insights gained could significantly inform the timing and targeting of GPC offerings to effectively mitigate the incidence of PPD, particularly in regions where teen parenting programs are being defunded [42,43].

Furthermore, future research should qualitatively investigate the perceptions of support and symptomatology of PPD across diverse cohorts of women. This inquiry will ensure that the symptom criteria incorporated in PPD screening instruments accurately reflect the experiences of women from varied age groups and backgrounds. Addressing this knowledge gap will enhance the understanding of PPD among all healthcare professionals in the outpatient setting, allowing for a more personalized approach to PPD screening and possibly directing PPD intervention efforts. Moreover, the findings from future PPD research should be used to aid in developing and pilot testing more culturally inclusive screening tools.

### 4.2. Strengths and Limitations

This study has several strengths, particularly in integrating social support into GPC. Social support was applied across four dimensions: emotional support, esteem support, social integration or network support, the provision of information and feedback support, and tangible assistance. The social support theory posits that, when all four dimensions of support are present, individuals can better cope with stressful life events [44]. Furthermore, the recruiting site has received certification from the Centering Healthcare Institute, which indicates fidelity in implementing GPC.

There are significant limitations regarding the study design. One limitation is that the intervention was not randomized. Participants’ choice to enroll in either GPC or IPC allowed for autonomy but may have also introduced selection bias. Furthermore, the study design limited us to examining associations and not causation. Additionally, our data were collected from a single site within one city and one obstetrical practice, which may affect the generalizability of the findings, especially when considering the applicability of our findings to other urban cities with larger Hispanic populations. Furthermore, a retrospective chart analysis ensured the study was adequately powered. Finally, all EPDS data are likely based on self-reports, which may be biased and influenced by social desirability.

## 5. Conclusions

Postpartum depression is a highly treatable mental health condition; however, Black and Hispanic women are the least likely to seek treatment. For decades, various barriers have persisted in their communities, preventing them from seeking help. Social support can act as a vital safety net, helping those who are undiagnosed and untreated for PPD avoid severe consequences, such as self-harm. In this study, social support integrated within GPC assisted the most at-risk women in averting severe symptoms associated with PPD. While GPC did not result in a statistically significant difference in EPDS scores, the study highlights the importance of further research to better understand PPD and identify effective ways to mitigate it. Thus, it is our position that GPC may be able to serve as a supportive intervention for maternal mental health.

## Figures and Tables

**Table 1:** Demographics and characteristics of the postpartum study participants (N = 200).

	N (%)	M (SD) Range
Type of Care Centering Individual	100 (50) 100 (50)	
Residence Adjacent Counties Baltimore City	60 (31.8) 137 (68.2)	
Maternal Race Black Hispanic	191 (95.5) 9 (4.5)	
Age 16–20 yrs 21–25 yrs 26–30 yrs 31–34 yrs 35 yrs and older	25 (12.6) 70 (35.2) 54 (27.1) 34 (17.1) 16 (8.0)	
Marital Status Single Married Divorced	164 (82) 36 (18.0) 0 (0.0)	
Prior History of Maternal Mental Health Condition No History Depression Anxiety	178 (89.4) 16 (8.0) 5 (10.6)	
BMI at Initial Prenatal Care Visit Weight in Pounds Initial Prenatal Care Visit		30.0 (8.1) 17.2–61.7 173 (48.6) 91–335 lbs.
History of Prior Births Prima Gravida 1 Previous Live Birth 2 or More Previous Live Births	93 (46.5) 58 (29.0) 49 (24.5)	
Prenatal Care Visits Attended		11 (2.6) 4–16
Type of Birth Spontaneous Vaginal Delivery Cesarean Birth Vacuum-Assisted Vaginal Delivery Vaginal Birth After Cesarean	121 (60.8) 64 (32.2) 10 (5.0) 4 (2)	
Gestational age at Birth		38.6 (1.7) 30.2–41.2 wks.
Length of Hospital Stay		2.7 (1.9) 0–19
Total Edinburgh Postnatal Depression Score		4.0 (4.6) 0- 33
Depression Risk Score Greater than 10 Score Less than 10	27(13.8) 168 (86.2)	
Depression Scores by Severity None–Minimum Mild Moderate Severe	147(75.4) 37 (19.0) 8 (4.1) 3 (1.5)	

Note: Data are missing on the following variables: history of maternal mental conditions, type of birth, Edinburgh Postnatal Depression Score, depression risk, and depression by severity. Note: lbs. = pounds, wks. = weeks.

**Table 2:** Participants’ responses to the Edinburgh Postnatal Depression Scale items.

	As Much as I Always Could N (%)	Not Quite So Much N (%)	Definitely Not So Much N (%)	Not at All N (%)
I have been able to laugh and see the funny side of things.	165 (83.8)	24 (12.2)	7 (3.6)	1 (0.5)
	As much as I ever did N (%)	Rather less than I used to N (%)	Definitely less than I used to N (%)	Hardly at all N (%)
I look forward to things with enjoyment.	158 (79.8)	36 (18.2)	3 (1.5)	1 (0.5)
	Yes, most of the time N (%)	Yes, some of the time N (%)	Not very often N (%)	No, never N (%)
* I blame myself unnecessarily when things went wrong.	5 (2.5)	31 (15.7)	51 (25.8)	111 (56.1)
	No, not at all N (%)	Hardley ever N (%)	Yes, sometimes N (%)	Yes, very often N (%)
I have been anxious or worried for no good reason.	118 (59.9)	37 (18.8)	32 (16.2)	10 (5.1)
	Yes, quite a lot N (%)	Yes, sometimes N (%)	No, not much N (%)	No, not at all N (%)
* I have felt scared or panicky for no good reason.	4 (2.0)	12 (6.1)	45 (22.7)	137 (69.2)
	Yes, most of the time I haven’t been able to cope N (%)	Yes, some of the time I haven’t been coping N (%)	No, most of the time I have coped quite well N (%)	No, I have been coping as well as ever N (%)
* Things have been getting on top of me.	3 (1.5)	31 (15.7)	61 (30.8)	103 (52.0)
	Yes, most of the time N (%)	Yes, sometimes N (%)	Not very often N (%)	No, not at all N (%)
* I have been so unhappy that I have had difficulty sleeping.	2 (1.0)	10 (5.1)	33(16.8)	152 (77.2)
	Yes, most of the time N (%)	Yes, quite often N (%)	No, not very often N (%)	No, not at all N (%)
* I have felt sad or miserable.	2 (1.0)	16 (8.1)	51 (25.8)	129 (65.2)
	Yes, most of the time N (%)	Yes, quite often N (%)	Only Occasionally N (%)	No, never N (%)
* I have been so unhappy that I’ve been crying.	4 (2.0)	8 (4.0)	47 (23.7)	139 (70.2)
	Yes, quite often N (%)	Sometimes N (%)	Hardley ever N (%)	Never N (%)
* The thought of harming myself has occurred to me.	0 (0.0)	2 (1.0)	5 (2.5)	191 (96.5)

Note: Items are scored 0–3. * The frequency may not sum due to missing data.

**Table 3:** Demographic and characteristics by the range of depression severity (N = 200).

	None/Minimum N (%)	Mild N (%)	Moderate N (%)	Severe N (%)	X^2^ Statistic	*p* Value N
Residence Adjacent Counties Inner-City Baltimore	44 (73.3) 103 (76.3)	11 (18.3) 26 (19.3)	4 (6.7) 4 (3.0)	1 (1.7) 2 (1.5)	X^2^ = 1.4	*p* = 0.690
Age 16–20 yrs 21–25 yrs 26–30 yrs 31–34 yrs 35 yrs and older	17 (70.8) 47 (69.1) 40 (75.5) 29 (87.9) 14 (87.5)	5 (20.8) 13 (19.1) 13 (24.5) 4 (12.1) 2 (12.5)	2 (8.3) 6 (8.8) 0 (0.0) 0 (0.0) 0 (0.0)	0 (0) 2 (2.9) 1 (1.9) 0 (0.0) 0 (0.0)	X^2^ = 14.3	*p* = 0.277
History of Maternal Mental Health No History Depression Anxiety	133 (76.4) 10 (66.7) 3 (60.0)	32 (18.4) 4 (26.7) 1 (20.0)	6 (3.4) 1 (6.7) 1 (20.0)	3 (1.7) 0 (0.0) 0 (0.0)	X^2^ = 4.66	*p* = 0.588
Married Status Married Single	27 (77.1) 120 (75)	6 (17.1) 31 (19.4)	2 (5.7) 6 (3.8)	0 (0.0) 3 (1.9)	X^2^ = 1.02	*p*= 0.797
History of Prior Births Prima Gravida 1 Previous Live Birth 2 or More Previous Live Births	67 (72.8) 42 (75.0) 38 (80.9)	18 (19.6) 11 (19.6) 8 (17.0)	5 (5.4) 2 (3.6) 1 (2.1)	2 (2.2) 1 (1.8) 0 (0.0)	X^2^ = 2.26	*p* = 0.894
Gestational Age at Birth Preterm < 37 weeks Term > 37 weeks	12 (66.7) 134 (76.1)	4 (22.2) 33 (18.0)	2 (11.1) 6 (3.4)	0 (0.0) 3 (1.7)	X^2^ = 2.95	*p* = 0.399

Note: Data are missing; some cells have fewer than expected counts.

**Table 4:** Comparison of characteristics by traditional and group prenatal care model (N = 200).

	Traditional Model	Group Model	Statistics	*p* Value
Number of Prenatal Care Visits	M (SD) 9.9 (2.6)	M (SD) 11.8 (2.3)	T = −5.39	*p* ≤ 0.001 **
Residence Adjacent Counties Inner-City Baltimore	N (%) 34 (34) 66 (66)	N (%) 29 (29) 71 (71)	X^2^ = 0.579	*p* = 0.447
Length of Hospital Stay	M (SD) 2.6 (1.8)	M (SD) 2.9 (1.9)	T = −1.12	*p* = 0.266
Age 16–20 yrs 21–25 yrs 26–30 yrs 31–34 yrs 35 yrs and older	N (%) 9 (9.0) 35 (35.0) 27(27.0) 19 (19.0) 10 (10.0)	N (%) 16 (16.0) 35 (35.0) 28 (28.0) 15 (15.0) 6 (6.0)	X^2^ = 3.45	*p* = 0.486
Married Status Married Single	N (%) 18 (18.0) 82 (82.0)	N (%) 18 (18.0) 82 (82.0)	X^2^ = 0.000	*p* = 1.0
History of Prior Births Prima Gravida 1 Previous Live Birth 2 or More Previous Live Births	N (%) 40 (40.0) 30 (30.0) 30 (30.0)	N (%) 53 (53.0) 28 (28.0) 19 (19.0)	X^2^ = 4.35	*p* = 0.113
History of Maternal Mental Health No History Depression Anxiety	N (%) 92 (92.9) 6 (6.1) 1 (1.0)	N (%) 86 (86.0) 10 (10.0) 4 (4.0)	X^2^ = 2.9	*p* = 0.223
Birth gestational age in weeks	M (SD) 38.61 (1.7)	M (SD) 38.63 (1.7)	T = 0.08	*p* = 0.704
Newborn Birth Weight in KG	M (SD) 3.11 (0.48)	M (SD) 3.37 (2.4)	T = −0.99	*p* = 0.274
EPDS Total Scores	M (SD) 3.8 (4.7)	M (SD) 4.3 (4.6)	T = 2.099	*p* = 0.461
EPDS at Risk for Depression EPDS score > 10 at risk EPDS score < 10 not at risk	N (%) 10 (10.3) 87 (89.7)	N (%) 17 (17.3) 81 (82.7)	X^2^ = 2.02	*p* = 0.155
EPDS by Severity None–Min Mild Moderate Severe	N (%) 76 (78.4) 16 (16.5) 2 (2.1) 3 (3.1)	N (%) 71 (72.4) 21 (21.4) 6 (6.1) 0 (0.0)	X^2^ = 5.8	*p* = 0.120

Note: Data are missing. Significance is denoted by ** *p* ≤ 0.001.

## Data Availability

The original contributions presented in this study are included in the article. Further inquiries can be directed to the corresponding authors.

## References

[1] Tabb K.M., Dalton V.K., Tilea A., Kolenic G.E., Admon L.K., Hall S.V., Zhang X., Ryckman K.K., Zivin K. (2023). Trends in Antenatal Depression and Suicidal Ideation Diagnoses among Commercially Insured Childbearing Individuals in the United States, 2008–2018. J. Affect. Disord..

[2] Carlson K., Mughal S., Azhar Y., Siddiqui W. (2025). Postpartum Depression. StatPearls.

[3] Agrawal I., Mehendale A.M., Malhotra R. (2022). Risk Factors of Postpartum Depression. Cureus.

[4] Saharoy R., Potdukhe A., Wanjari M., Taksande A.B. (2023). Postpartum Depression and Maternal Care: Exploring the Complex Effects on Mothers and Infants. Cureus.

[5] Floyd James K., Smith B.E., Robinson M.N., Thomas Tobin C.S., Bulles K.F., Barkin J.L. (2023). Factors Associated with Postpartum Maternal Functioning in Black Women: A Secondary Analysis. J. Clin. Med..

[6] Khadka N., Fassett M.J., Oyelese Y., Mensah N.A., Chiu V.Y., Yeh M., Peltier M.R., Getahun D. (2024). Trends in Postpartum Depression by Race, Ethnicity, and Prepregnancy Body Mass Index. JAMA Netw. Open.

[7] Meekins C. (2022). Supporting Black Women’s Maternal Mental Health Journey.

[8] McGovern M.E., Rokicki S., Von Jaglinsky A., Reichman N.E. (2023). Neighborhood-Level Housing Affordability and Maternal Depression. SSM-Ment. Health.

[9] Tsai W.-L., Nash M.S., Rosenbaum D.J., Prince S.E., D’Aloisio A.A., Mehaffey M.H., Sandler D.P., Buckley T.J., Neale A.C. (2023). Association of Redlining and Natural Environment with Depressive Symptoms in Women in the Sister Study. Environ. Health Perspect..

[10] Andris C. (2024). Social Life and Interpersonal Relationships in Environment and Planning B. Environ. Plan. B Urban Anal. City Sci..

[11] Mouratidis K., Poortinga W. (2020). Built Environment, Urban Vitality and Social Cohesion: Do Vibrant Neighborhoods Foster Strong Communities?. Landsc. Urban Plan..

[12] Sheikh J., Allotey J., Kew T., Fernández-Félix B.M., Zamora J., Khalil A., Thangaratinam S., Abdollahain M., Savitri A.I., Salvesen K.Å. (2022). Effects of Race and Ethnicity on Perinatal Outcomes in High-Income and Upper-Middle-Income Countries: An Individual Participant Data Meta-Analysis of 2 198 655 Pregnancies. Lancet.

[13] Ahad A.A., Sanchez-Gonzalez M., Junquera P. (2023). Understanding and Addressing Mental Health Stigma Across Cultures for Improving Psychiatric Care: A Narrative Review. Cureus.

[14] Williams D.R., González H.M., Neighbors H., Nesse R., Abelson J.M., Sweetman J., Jackson J.S. (2007). Prevalence and Distribution of Major Depressive Disorder in African Americans, Caribbean Blacks, and Non-Hispanic Whites. Arch. Gen. Psychiatry.

[15] Place J.M.S., Renbarger K., Van De Griend K., Guinn M., Wheatley C., Holmes O. (2024). Barriers to Help-Seeking for Postpartum Depression Mapped onto the Socio-Ecological Model and Recommendations to Address Barriers. Front. Glob. Womens Health.

[16] Silva S., Canavarro M.C., Fonseca A. (2018). Why Women Do Not Seek Professional Help for Anxiety and Depression Symptoms during Pregnancy or throughout the Postpartum Period? Barriers and Facilitators of the Help-Seeking Process. Psychol. Pract. Res. J..

[17] Trost S.L., Beauregard J.L., Smoots A.N., Ko J.Y., Haight S.C., Moore Simas T.A., Byatt N., Madni S.A., Goodman D. (2021). Preventing Pregnancy-Related Mental Health Deaths: Insights From 14 U.S. Maternal Mortality Review Committees, 2008–2017. Health Aff..

[18] Shrivastava S.R., Shrivastava P.S., Ramasamy J. (2015). Antenatal and Postnatal Depression: A Public Health Perspective. J. Neurosci. Rural Pract..

[19] Sampson M., Yu M., Mauldin R., Mayorga A., Gonzalez L.G. (2021). ‘You Withhold What You Are Feeling so You Can Have a Family’: Latinas’ Perceptions on Community Values and Postpartum Depression. Fam. Med. Community Health.

[20] Floyd James K., Reaves K., Richards M.C., Choi K.R. (2024). Enhancing Obstetric Healthcare Providers’ Knowledge of Black Maternal Mental Health: A Feasibility Study. Int. J. Environ. Res. Public Health.

[21] Buultjens M., Farouque A., Karimi L., Whitby L., Milgrom J., Erbas B. (2021). The Contribution of Group Prenatal Care to Maternal Psychological Health Outcomes: A Systematic Review. Women Birth.

[22] Gennaro S., Melnyk B.M., Szalacha L.A., Gibeau A.M., Hoying J., O’Connor C.M., Cooper A.R., Aviles M.M. (2024). Effects of Two Group Prenatal Care Interventions on Mental Health: An RCT. Am. J. Prev. Med..

[23] Lenze S.N., McKay-Gist K., Paul R., Tepe M., Mathews K., Kornfield S., Phillips C., Smith R., Stoermer A., Carter E.B. (2024). Elevating Voices, Addressing Depression, Toxic Stress, and Equity Through Group Prenatal Care: A Pilot Study. Health Equity.

[24] Al-Mutawtah M., Campbell E., Kubis H.-P., Erjavec M. (2023). Women’s Experiences of Social Support during Pregnancy: A Qualitative Systematic Review. BMC Pregnancy Childbirth.

[25] Renbarger K.M., Place J.M., Schreiner M. (2021). The Influence of Four Constructs of Social Support on Pregnancy Experiences in Group Prenatal Care. Women’s Health Rep..

[26] Byerley B.M., Haas D.M. (2017). A Systematic Overview of the Literature Regarding Group Prenatal Care for High-Risk Pregnant Women. BMC Pregnancy Childbirth.

[27] Tessema M., Abera M., Birhanu Z. (2024). Effectiveness of Group-Based Psycho-Education on Preventing Postpartum Depression among Pregnant Women by Primary Healthcare Provider in Primary Healthcare Institution: A Cluster-Randomized Controlled Trial. Front. Psychiatry.

[28] Short V.L., Hand D.J., Mancuso F., Raju A., Sinnott J., Caldarone L., Rosenthall E., Liveright E., Abatemarco D.J. (2024). Group Prenatal Care for Pregnant Women with Opioid Use Disorder: Preliminary Evidence for Acceptability and Benefits Compared with Individual Prenatal Care. Birth.

[29] Felder J.N., Epel E., Lewis J.B., Cunningham S.D., Tobin J.N., Rising S.S., Thomas M., Ickovics J.R. (2017). Depressive Symptoms and Gestational Length among Pregnant Adolescents: Cluster Randomized Control Trial of Centering Pregnancy® plus Group Prenatal Care. J. Consult. Clin. Psychol..

[30] Abell Foundation Baltimore Green Space. https://abell.org/news-and-features/baltimore-green-space/.

[31] Baltimore County Government Baltimore County Releases Vacant Properties Portal. https://www.baltimorecountymd.gov/county-news/2023/11/21/baltimore-county-releases-vacant-properties-portal.

[32] Miller M., McComas M. (2022). The Costs of Baltimore’s Vacant Housing. https://abell.org/wp-content/uploads/2022/08/The-Costs-of-Baltimores-Vacant-Housing.pdf.

[33] Manant A., Dodgson J.E. (2011). Centering Pregnancy: An Integrative Literature Review. J. Midwifery Womens Health.

[34] Cox J., Holden J., Henshaw C. (2014). Perinatal Mental Health: The Edinburgh Postnatal Depression Scale (EPDS) Manual.

[35] Regmi S., Sligl W., Carter D., Grut W., Seear M. (2002). A Controlled Study of Postpartum Depression among Nepalese Women: Validation of the Edinburgh Postpartum Depression Scale in Kathmandu. Trop. Med. Int. Health.

[36] McCabe-Beane J.E., Segre L.S., Perkhounkova Y., Stuart S., O’Hara M.W. (2016). The Identification of Severity Ranges for the Edinburgh Postnatal Depression Scale. J. Reprod. Infant Psychol..

[37] The United Census Bureau Quick Facts Baltimore City, Maryland. https://www.census.gov/quickfacts/fact/table/baltimorecitymaryland/INC110223.

[38] Maryland Health Department Live Birth Data 2022. https://health.maryland.gov/vsa/Documents/Reports%20and%20Data/Live%20Births/Live_Birth_2022.pdf.

[39] Bradshaw H., Riddle J.N., Salimgaraev R., Zhaunova L., Payne J.L. (2022). Risk Factors Associated with Postpartum Depressive Symptoms: A Multinational Study. J. Affect. Disord..

[40] Perez N.B., D’Eramo Melkus G., Vorderstrasse A.A., Wright F., Yu G., Sun Y.V., Crusto C.A., Taylor J.Y. (2022). Latent Class Analysis of Depressive Symptom Phenotypes Among Black/African American Mothers. Nurs. Res..

[41] Hymas R., Girard L.-C. (2019). Predicting Postpartum Depression among Adolescent Mothers: A Systematic Review of Risk. J. Affect. Disord..

[42] Maryland Department of Health: Maternal and Child Health Bureau Maryland Child and Adolescent Program. https://health.maryland.gov/phpa/mch/Pages/child_health.aspx.

[43] Trotman G., Chhatre G., Darolia R., Tefera E., Damle L., Gomez-Lobo V. (2015). The Effect of Centering Pregnancy versus Traditional Prenatal Care Models on Improved Adolescent Health Behaviors in the Perinatal Period. J. Pediatr. Adolesc. Gynecol..

[44] Drageset J. (2021). Social Support. Health Promotion in Health Care—Vital Theories and Research.

